# Harnessing adult-plant resistance genes to deploy durable disease resistance in crops

**DOI:** 10.1042/EBC20210096

**Published:** 2022-09-30

**Authors:** Eric Dinglasan, Sambasivam Periyannan, Lee T. Hickey

**Affiliations:** 1 Queensland Alliance for Agriculture and Food Innovation, The University of Queensland, Brisbane, QLD, Australia; 2 Black Mountain Science and Innovation Park, CSIRO Agriculture and Food, Canberra, ACT, Australia

**Keywords:** adult plant resistance, breeding technologies, cereals, genebanks, plant breeding, rust

## Abstract

Adult-plant resistance (APR) is a type of genetic resistance in cereals that is effective during the later growth stages and can protect plants from a range of disease-causing pathogens. Our understanding of the functions of *APR*-associated genes stems from the well-studied wheat-rust pathosystem. Genes conferring APR can offer pathogen-specific resistance or multi-pathogen resistance, whereby resistance is activated following a molecular recognition event. The breeding community prefers APR to other types of resistance because it offers broad-spectrum protection that has proven to be more durable. In practice, however, deployment of new cultivars incorporating APR is challenging because there is a lack of well-characterised APRs in elite germplasm and multiple loci must be combined to achieve high levels of resistance. Genebanks provide an excellent source of genetic diversity that can be used to diversify resistance factors, but introgression of novel alleles into elite germplasm is a lengthy and challenging process. To overcome this bottleneck, new tools in breeding for resistance must be integrated to fast-track the discovery, introgression and pyramiding of *APR* genes. This review highlights recent advances in understanding the functions of *APR* genes in the well-studied wheat-rust pathosystem, the opportunities to adopt *APR* genes in other crops and the technology that can speed up the utilisation of new sources of APR in genebank accessions.

## Introduction

In cereals, rust diseases caused by the fungus *Puccinia* spp. are among the primary threats to future crop production. Globally, yield losses due to diseases, including rust, are managed using genetic resistance, which is the innate ability of plants to resist pests and diseases. In general, genes conferring resistance to rust diseases fall into two broad categories: all-stage resistance (ASR) and adult-plant resistance (APR). ASR, the predominant type, refers to resistance beginning at the seedling phase and continuing throughout all crop growth stages. *ASR* genes function only against selected strains of a pathogen, as recognition of effector molecules specific to these strains is a prerequisite. Hence, *ASR* genes are also referred to as seedling or race-specific resistance genes. While most *ASR* genes remain effective throughout all stages of development, there are exceptions, such as the barley leaf rust resistance gene *Rphq2* which is ineffective at adult growth stages [1]. Adult-plant resistance (APR) refers to resistance against pathogen infection acting only during adult growth stages. Generally, *APR* genes protect plants from a wide range of pathogen strains and confer race-nonspecific resistance. However, there are a few exceptions where *APR* genes confer race-specific resistance [2]. A characteristic feature of most APRs is their partial resistance nature which allows the pathogen to sporulate at a reduced or slower rate. Therefore, APRs are often considered more durable because individually they exert lower selection pressure for mutations in the pathogen population that overcome the resistance [3]. Notably, some well-known *APR* genes, such as *Leaf rust resistance* (*Lr*)*34* and *Lr67* of wheat (*Triticum aestivum*), have pleiotropic functions: they are effective against the three rusts (stem, leaf and stripe) and powdery mildew [4]. Therefore, *APR* genes can be broadly subclassified into two groups: pathogen-specific resistance and multi-pathogen resistance.

Modern plant breeding has successfully created the highly productive crops that we grow today. However, a combination of intensive selection and the release of cultivars with single or few resistance genes has unintentionally constrained the diversity of effective resistance genes in elite germplasm, posing a major breeding challenge for future sustainable crop production. For rapidly evolving pathogens, such as rusts, and to avoid resistance breakdown, broad-spectrum and durable resistance offers a more sustainable control strategy. This approach necessitates holistic and efficient genetic control, as well as the discovery of more broad-spectrum resistance factors. Germplasm collections preserved in genebanks worldwide provide a great source of genetic variation that can be used to diversify resistance factors in the modern gene pool. Traditionally, this was a tedious and challenging undertaking. However, new breeding tools now allow novel resistance alleles to be rapidly discovered and introgressed into adapted germplasm.

## APR: the wheat-rust pathosystem

### Pathogen-specific *APR* genes

**Figure 1:**
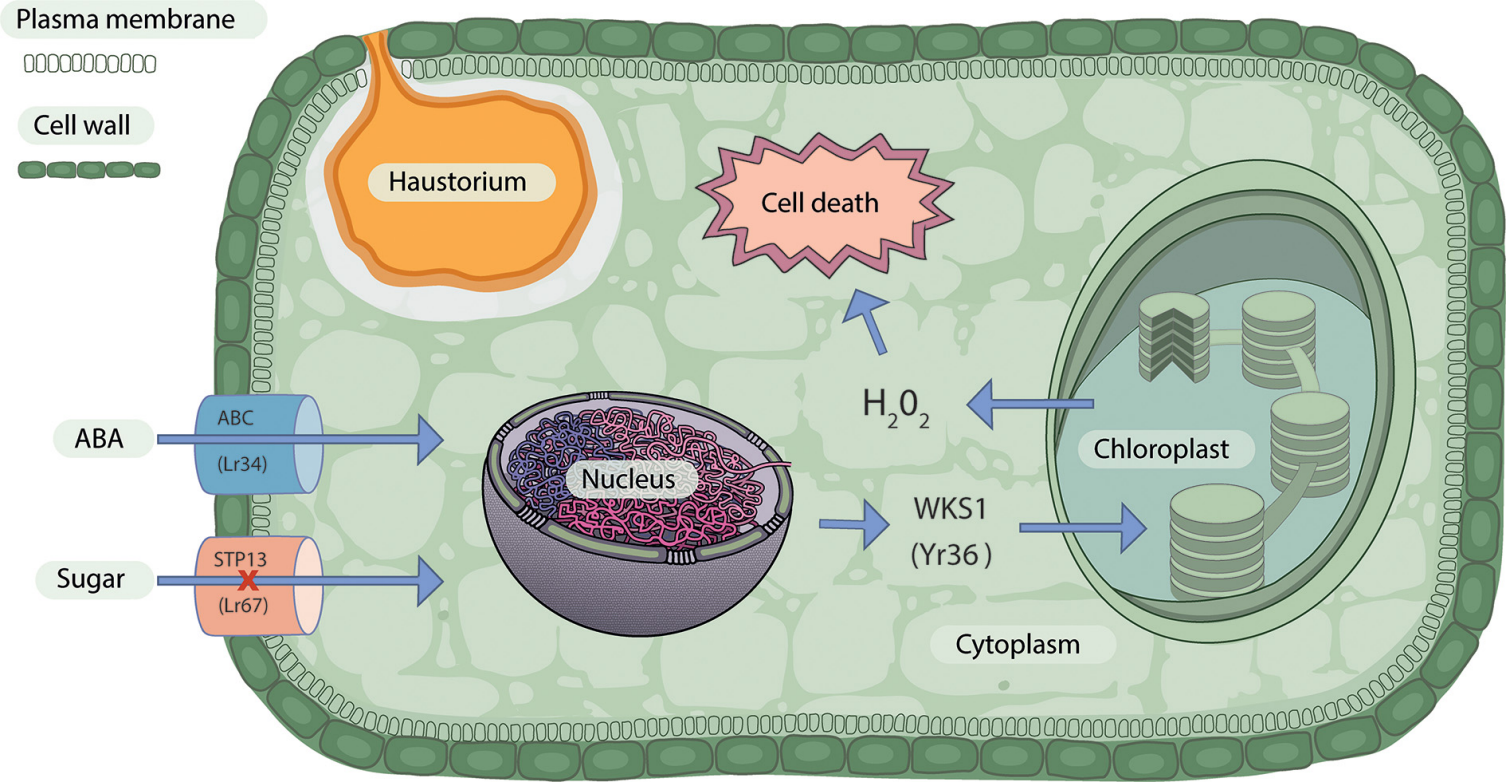
Mechanistic model of *APR* gene function Pathogen-specific (*Yr36*) and multi-pathogen (*Lr34* and *Lr67*) *APR* genes and their involvement in plant cell signalling and defence pathways. Yr36, a wheat kinase START1 (WKS1) protein, mediates resistance to wheat stripe rust through phosphorylation of photosystem II related proteins in the chloroplast and production of reactive oxygen species and H_2_O_2_. Lr34 and Lr67 are adenosine triphosphate-binding cassette (ABC) and sugar transporter (STP) proteins that confer multi-pathogen resistance through regulation of abscisic acid (ABA) and hexose sugar molecules, respectively.

Pathogen-specific *APR* genes are effective against diverse strains of a specific rust pathogen. For example, *Yellow rust resistance* (*Yr*)*36*, an *APR* gene derived from the tetraploid emmer wheat (*Triticum turgidum* ssp. *dicoccoides*), provides resistance to diverse strains of stripe rust but is ineffective against leaf or stem rust. *Yr36* encodes a protein that has a serine/threonine kinase and steroidogenic acute regulatory protein-related lipid-transfer (START) domain; the gene is therefore also known as *Wheat Kinase START1* (*WKS1*) [5]. WKS1 mediates resistance through phosphorylation of the photosystem II manganese-stabilising polypeptide (psbO) protein complex present in the thylakoid membrane of the chloroplast (Figure 1). Phosphorylation of psbO leads to the production of reactive oxygen species and eventually H_2_O_2_, which induces cell-death-mediated defence against the stripe rust fungus. Subsequently, WKS1 phosphorylates the enzyme thylakoid ascorbate peroxidase (tAPX) to prevent degradation of H_2_O_2_ [6,7].

In a homology search, *WKS1*-like sequences were also detected in wild grass species, such as *Aegilops longissimi*, *Dasyprum villosum*, *Lophopyrum elongatum*, *Pseudoroegneria gracillima* and *Thinopyrum bessarabicum.* However, there were numerous gene sequences in the rice (*Oryza sativa*) and Arabidopsis (*Arabidopsis thaliana*) genomes encoding either the WKS1 START1 or kinase domains, but hardly any sequences encoding both domains. Hence, the fusion of START1 and the kinase domain to form the functional *WKS1* gene was a recent evolutionary event that occurred after the divergence of wheat and its related grass species from rice. The Arabidopsis genome contains the *enhanced disease resistance* (*EDR2*) gene, which is related to *WKS1.* While EDR2 has a START1 domain similar to that of WKS1, it lacks the kinase domain and is a negative regulator of defence, promoting infection by *Golovinomyces cichoracearum*, a powdery mildew pathogen. On the other hand, the kinase domain of WKS1 is related to the wall-associated kinase (WAK) proteins, which are also known to play a substantial role in defence against pathogens in plants [5].

Similar to *WKS1* resistance, new loci conferring pathogen-specific APR for leaf, stem and stripe rust were identified from the large-scale screening of wheat landrace collections, including the Watkins and N.I. Vavilov Institute of Plant Genetic Resources (VIR) wheat collections. Through rust screening and genotyping the collections using either the 90K single-nucleotide polymorphism (SNP) or diversity array technology (DArT) platforms, genome-wide association studies (GWAS) enabled the rapid discovery of new quantitative trait loci (QTLs) associated with APR [8–11]. The recently established gold standard reference genomes of cultivated wheat and its progenitors will accelerate the identification of markers and candidate genes associated with these QTLs [12].

### APR against multiple isolates and pathogens

#### Lr34-mediated multi-pathogen resistance

The *Lr34* locus with *Lr34/Yr18/Stem rust resistance* (*Sr*)*57/Powdery mildew resistance* (*Pm*)*38/Spot blotch (Sb)1* resistance is one of wheat’s earliest known and most widely deployed multi-pathogen resistance genes [13]. Soon after its initial identification in the early 20th century, the gene was used extensively in wheat cultivation. A unique characteristic of these pleiotropic functional genes is their association with Leaf Tip Necrosis (LTN), i.e., senescence around the tip of the flag leaf, which is widely used as a visual marker to select these genes in breeding programs. An adenosine triphosphate-binding cassette (ABC) transporter was identified as the key gene responsible for *Lr34*-mediated resistance through map-based cloning (Figure 1). The ABC protein is part of the pleiotropic drug resistance (PDR) transporter family, common in plants and fungi but absent in the animal genome. The Arabidopsis and rice genomes encode 15 and 23 PDR proteins, respectively, all of which have uniform structural features with a pair of cytosolic and transmembrane domains. PDR proteins confer resistance to chemicals such as fungicides, herbicides, pesticides, antibiotics and detergents. Specifically, in plants, PDR proteins play a significant role in biotic and abiotic stress tolerance [14]. For instance, like the wheat ABC transporter, the *Penetration3* (*PEN3*, also known as *PDR8*) gene in Arabidopsis is also known for signalling defence against pathogens as it provides non-host resistance to powdery mildew in barley (*Hordeum vulgare*) and pea (*Pisum sativum*), potato late blight and cabbage root rot diseases [15,16].

Following the identification of an ABC transporter as being responsible for *Lr34*-mediated resistance, the *Lr34* gene sequence from wheat was introduced into other cereals such as barley, which rarely carries a functional ortholog of *Lr34*. As in wheat, transgenic barley lines carrying *Lr34* showed resistance against multiple diseases, such as wheat stem rust, barley leaf rust and powdery mildew. Additionally, the *Lr34*-carrying barley lines showed the LTN phenotypes observed in wheat. An exciting feature in barley is that the transgene *Lr34* is expressed and functional at a very early (seedling) growth stage, unlike in wheat, where it is active only at the later growth stages [17]. As a transgene, wheat *Lr34* is also functional in rice, where it confers resistance to blast disease from the seedling growth stage. With its defence against rice blast disease, the *Lr34* gene’s function is extended beyond the biotrophs (rust and mildew), as *Magnaporthe oryzae*, the causative agent of rice blast disease, is a hemibiotroph [18]. Likewise, *Lr34* provides resistance against both biotrophic (rust) and hemibiotrophic (anthracnose) pathogens in sorghum (*Sorghum bicolor*) [19] and reduces the incidence of rust and northern corn leaf blight disease in maize (*Zea mays*) [20].

Because *Lr34* from hexaploid wheat is functional as a transgene in other cereals, such as rice and barley, these diploid grasses with small genomes and fully annotated gene sets serve as a robust system for the in-depth investigation of *Lr34* function. For instance, using transgenic rice lines with varying expression levels of *Lr34* and transcriptome analysis (RNA-seq), expression levels of genes related to abscisic acid (ABA) regulatory pathways were found to correlate with *Lr34* expression. Subsequently, through *in planta* and *in vitro* yeast assays, ABA was identified as a key substrate for the ABC transporter in its induction of *Lr34-*mediated disease resistance. The role of ABA was further confirmed through physiological tests showing that *Lr34*-carrying lines had elevated tolerance of abiotic stress [21]. Surprisingly, the total content of ABA in the leaves of *Lr34*-carrying lines did not differ significantly from that of control lines. However, more ABA was detected at the leaf tip, compared with the basal or midrib portion, in the leaves of the resistant lines. The tissue-specific dispersal of ABA molecules towards the leaf tip is in line with the LTN phenotype associated with *Lr34*. Further, this trend of association between the tissue-specific distribution of ABA and disease resistance is evident from the expression of *Pathogenesis Related* (*PR*) gene members (*PR1*, *PR3*, *PR5*, *PR9* and *PR10*) at the leaf tip [22].

#### Lr67-mediated multi-pathogen resistance

Following the widespread selection and deployment of *Lr34* in wheat breeding programs, genes with similar pleiotropic functions were screened vigorously for commercial crop protection. The other well-known example in this category is the *Lr67* locus (*Lr67/Yr46/Sr55/Pm46*), which, like *Lr34*, also confers resistance to leaf, stripe and stem rust and powdery mildew diseases of wheat. However, a sugar transporter protein (STP) belonging to the sub-group STP13 is responsible for *Lr67*-mediated multi-pathogen resistance (Figure 1). Genes encoding STP13 proteins are conserved among plant species and are known widely for involvement in pathogen defence, as reported in Arabidopsis and grapevine (*Vitis vinifera*). Notably, Lr67 is a mutant version of STP, whose ability to transport glucose molecules from the apoplast to the cytosol is restricted. *Lr67*, located in the D genome of hexaploid wheat, has a dominant-negative effect whereby it interferes with the sugar transport function of the two *Lr67*-like pathogen susceptibility genes present in the A and B genomes. However, in Arabidopsis, increased uptake of sugar molecules by the *A. thaliana* (At)STP13 protein induces resistance against *Pseudomonas* spp. bacterial and grey mould fungal pathogens [23,24].

*H. vulgare* (*Hv*)*STP13* is similar to the pathogen-susceptible version of wheat *Lr67* in that it also encodes a protein that is involved in the transport of glucose molecules. As pathogens are sensitive to the disturbance of these sugar transporter functions, powdery-mildew-resistant barley lines were generated recently by mutating *HvSTP13* [25]. Subsequently, introduction of wheat *Lr67* into barley was found to perturb the functioning of *HvSTP13*, as the resulting transgenic lines were resistant to barley leaf rust and powdery mildew diseases. Similar to *Lr34* in barley, this resistant version of *HvSTP13* is expressed early in plant development in the transgenic barley lines, and its protein product activates *PR* genes to induce defence. This seedling function could be due to elevated gene expression in the diploid barley genome compared with the more complex wheat hexaploid genome [24]. Like barley, the model legume *Medicago truncatula* carries an *STP13* gene (*MtSTP13.1*) that is related to the pathogen-susceptible version of wheat *Lr67.* Fascinatingly, the conversion of *MtSTP13.1* to a pathogen-resistant version similar to *Lr67*, through mutations resulting in a G144R amino acid substitution, leads to resistance against powdery mildew disease. This important study now extends the application of *Lr67* function to dicot crops, such as legumes, where disease is a major constraint to meeting future production demand [26].

### APR to pathogens other than rusts

In addition to resistance against biotrophic fungi, such as rust and powdery mildew, studies have reported the involvement of *APR* genes in resistance against hemibiotrophic and necrotrophic diseases, such as tan spot [27], *Septoria nodorum* blotch [28] and *Fusarium* head blight/crown rot [29] of wheat. Outside the cereals, *APR* genes are also known globally for their involvement in resistance against important diseases of other crops, such as maize northern leaf spot [30], canola blackleg [31], *Brassica* downy mildew [32], chickpea *Fusarium* wilt and *Ascochyta* blight [33], and soybean powdery mildew [34]. Beside conveying resistance against multiple pathogen strains, these *APR* genes play a vital role in prolonging the durability of frequently used major or seedling resistance genes when deployed along with them. Although the QTLs or genomic regions conferring these resistance traits have been identified through GWAS and comparative genomic analysis, many of the underlying causal genes are unknown. This hampers our ability to unravel the underlying molecular mechanisms of these high-value resistance genes [31]. Only in the case of maize, the APR gene *Helminthosporium maydis (Hm)1* for northern leaf spot was identified to encode an NADPH-dependent reductase. The gene confers resistance through inactivation of the HC-toxin produced by the pathogen, *Cochliobolus carbonum* [30].

### New sources of APR and breeding strategies

#### Challenges associated with breeding for APR

In the face of rapidly evolving pathogens, there is a need to diversify resistance factors to combat future disease epidemics and achieve the full potential of new cultivars. For example, in Australia, several elite wheat cultivars that are high yielding, with good quality and agronomic traits, remain susceptible to rust diseases [35]. Intensive selection in modern breeding programs has restricted the genetic variation of elite germplasm [36,37], which also presents a bottleneck for sustained resistance breeding into the future. In the past, breeders unknowingly deployed a small number of genes or a single gene in numerous cultivars, which increased the risk of genetic vulnerability and reduced diversity for resistance factors [38–40,3]. For this reason, APR has become a major target for cereal breeding programs because it offers a more durable and constitutive broad-spectrum protection against multiple diseases. Initially, only a handful of well-characterised *APR* genes were available to breeders, who therefore strived to combine them with *ASR* genes already present in elite breeding materials. There are many uncharacterised *APR* genes in elite populations that breeders can combine. However, this is particularly challenging due to many reasons, including the lack of linked molecular markers to support selection, poor knowledge of their phenotypic effects and gene interactions, and poor knowledge of their environmental stability and genetic background effects. As the preference for APR strengthens in the breeding community, there is a growing demand for identifying and characterising a larger arsenal of *APR* genes in elite germplasm.

#### Genebank collections provide valuable sources of APR

Exploiting genetic variation has become a common theme in diversifying the disease-resistance traits of the modern gene pool [41]. Diverse genebank accessions are commonly used for the introgression of disease-resistance genes that are absent in modern germplasm. Efforts to preserve genetic diversity stem from historical epidemics of plant diseases impacting food crops, together with a recognition of the importance of genebank collections in plant breeding. There are 1,750 genebanks worldwide that contain genetic diversity of approximately 7.4 million accessions representing more than 16,500 plant species [42]. For wheat, 80 genebanks storing over 800,000 accessions have been established globally [43]. The largest wheat collection, with 110,000 accessions, is currently maintained at the International Maize and Wheat Improvement Center (CIMMYT) in Mexico [44].

Tapping into the genetic diversity of these collections can help to diversify resistance alleles in elite breeding populations and improve resistance levels of future cultivars [45–46]. Several studies demonstrate that genebanks provide a good source of genetic variation for APR. For instance, GWAS using wheat landraces and historical breeding lines from the VIR identified novel QTLs for APR to leaf rust [9], tan spot [47] and stripe rust [11]. The Watkins Collection, comprising landraces from different countries, provided novel sources of APR to leaf rust, stripe rust and stem rust [48]. Chinese landraces from the National Germplasm Repository of the Chinese Academy of Agricultural Sciences also provided sources of APR to stripe rust [49,50]. A Mexican core set of wheat accessions representing the complete variation of hexaploid landraces was found to include novel APR to both yellow rust and stem rust [51]. In addition to landraces, crop wild relatives, such as wild emmer wheat (*T. turgidum*), are also reported to carry novel sources of APR to stripe rust [52].

Despite the promise of diverse sources of APR, the use of unadapted germplasm comes with many challenges. Introgression of novel genes using traditional backcrossing or marker-assisted selection approaches has been implemented in many breeding programs [53] but is usually successful only for traits with simple genetic control [54]. While gene pyramiding through biotechnology-based approaches and marker-assisted breeding has proven effective for resistance breeding, and these technologies have several advantages, they are restricted to a small number of genes or QTLs [55]. The main difficulty in using genebank accessions, such as landraces, arises from, in theory, the need to maximise introgression of large numbers of genes or QTLs linked to APR with small effects while avoiding linkage drag or pleiotropy during the backcrossing process.

At CIMMYT, significant progress has been achieved towards developing high-yielding wheat varieties with high levels of APR to multiple diseases such as leaf rust, stem rust, stripe rust and powdery mildew [40]. Researchers employed a shuttle breeding scheme and targeted crossing program, wherein wheat materials identified to carry APR were crossed with high-yielding materials and the offspring underwent intensive selection and field testing.

#### New technologies to support breeding for APR

Traditionally, plant breeders evaluate segregating plant populations in the field for APR using specialised disease screening nurseries. Using this approach in a breeding program can accumulate *APR* genes and slowly improve resistance levels over time. However, technologies are rapidly evolving and can be applied to speed up resistance breeding. For instance, speed breeding can significantly reduce generation time and fast-track both pre-breeding and breeding goals [56]. By controlling the photoperiod and temperature conditions, speed breeding enables growing up to six plant generations of major crops, such as wheat and barley [56,57]. Thus, speed breeding can provide a useful tool to accelerate the development of QTL mapping populations to enable faster discovery of new *APRs* [27]. Identification of molecular markers linked to *APRs* can also assist introgression into elite germplasm and subsequent gene stacking. By combining marker-assisted selection and speed breeding in spring wheat, it is possible to transfer and combine multiple APRs into any elite genetic background within 18 months.

To accelerate phenotyping, protocols in wheat have been successfully adapted to speed breeding conditions, which permit rapid screening for APR all year round [58–60]. Application of the rapid screening approach has accelerated the discovery of APR QTLs in a diverse landrace collection [9,11,47]. Imaging techniques coupled with machine learning could also enable precise and large-scale evaluation of APR phenotypes in both controlled and field environments [61]. Phenotyping can also be supported by fungal biomass quantification-based assays where variation among resistant and susceptible lines could be used as a proxy. This strategy could be useful where resistance levels are difficult to assess visually and could assist selection for genes with minor effect. For example, quantitative polymerase chain reaction (qPCR)-based assays were integrated with traditional visual assessments to assist in the selection of crown rot resistance in wheat and barley [62].

With low-cost and reliable genotyping platforms now available, genomic selection (GS) is being adopted in many crop breeding programs. It is especially advantageous in predicting the breeding values of candidate lines without phenotypic information, using only genomic information inferred from the prediction models of the training population [63]. GS has been successfully applied to identify high-merit lines with resistance to wheat stem rust, yellow rust, *Fusarium* head blight, *Septoria tritici* blotch, *Septoria nodorum* blotch and tan spot [64–73]. While disease resistance is important, in a breeding program many traits must be improved simultaneously. GS offers a flexible framework to apply weighting to multiple traits [74], including APR, quality and yield traits, to improve breeding populations over time.

In the context of GWAS, which are typically applied to diversity collections or breeding populations, single marker-trait associations are limited because a marker may not remain in linkage disequilibrium with the causal gene in subsequent generations of breeding [75]. To overcome this challenge, breeders can target the local linkage disequilibrium or haplotype [76] to facilitate introgression and stacking of APR QTLs. Using diverse collections, haplotype-based analysis according to local genomic estimated breeding values [77] offers another approach to discover favourable haploblocks conferring APR. However, once haploblocks are defined the problem becomes which plants to select as parents to stack large numbers of desirable haploblocks in the shortest timeframe possible. One promising strategy involves the use of artificial intelligence genetic algorithms to assist parent selection [78]. With artificial-intelligence-based selection, haploblocks in linkage drag with undesirable traits can be avoided and only the favourable haplotypes are introduced. Computer simulations to create digital twins of different crossing scenarios can provide decision-making tools to make efficient and targeted crossing designs. By combining this approach with speed breeding, the resistance haploblocks can then be introgressed and stacked to rapidly develop new populations with improved resistance. This provides a new framework for resistance breeding programs to harness big data by integrating technologies such as artificial intelligence, computational breeding and speed breeding.

## Outlook for cloning and engineering APR genes

**Figure 2:**
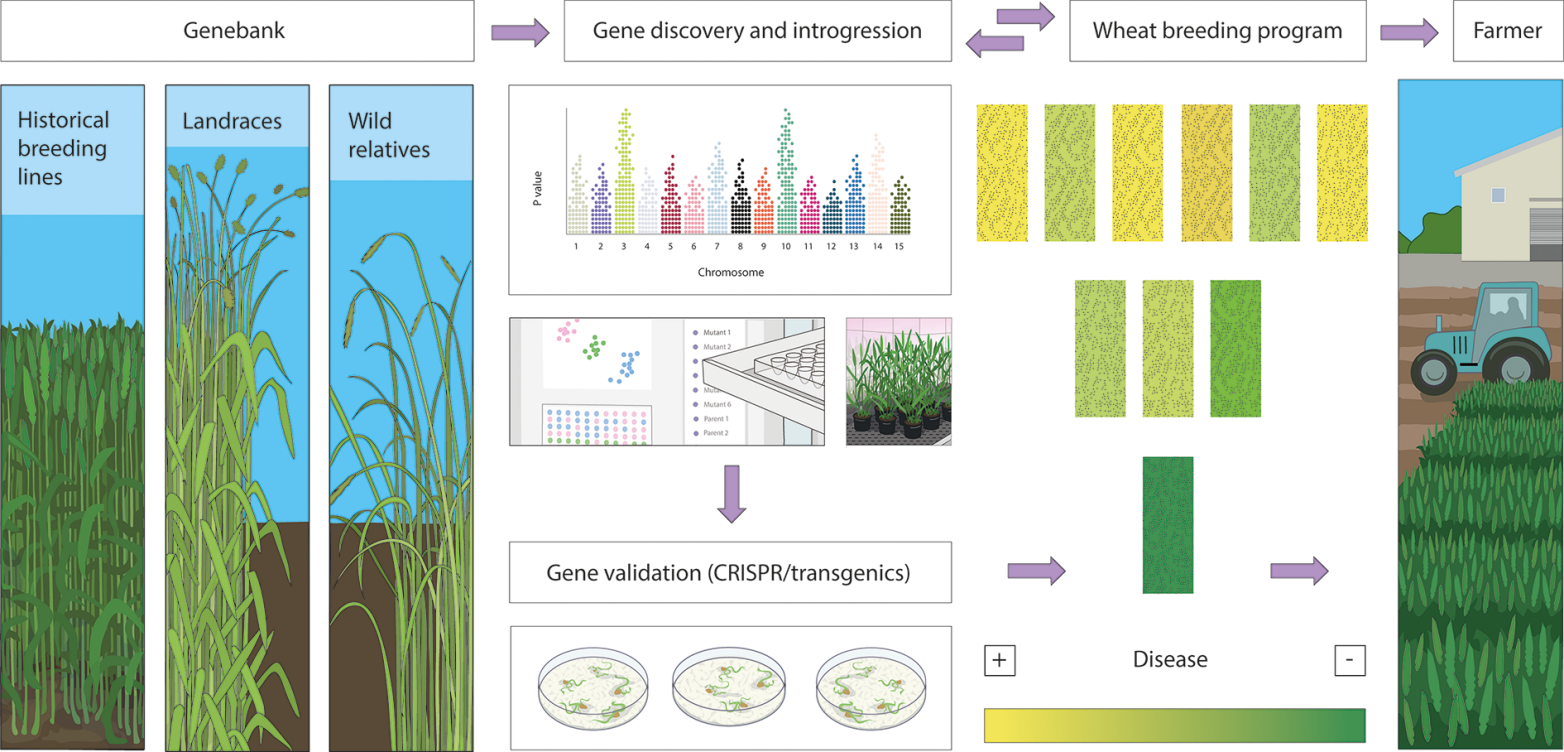
The path from genebank to deployment of APR into new wheat cultivars Genebank accessions such as historical breeding lines, landraces and wild relatives provide a rich source of genetic variation, including APR. Researchers can use a range of breeding technologies can accelerate the discovery of *APRs* in genebank accessions and introgress them into elite germplasm. Next, the *APRs* must be combined with other traits in a breeding program to develop disease resistant, high-yielding, and high-quality wheat varieties for farmers.

In crops with large and polyploid genomes, advances in sequencing technology and new complexity reduction approaches (e.g., targeted gene capture and chromosome-isolation-based strategies) will continue to accelerate the discovery of candidate *APR* genes [79]. Following discovery, researchers can now functionally characterise resistance gene candidates in model organisms, such as yeast. For example, resistance genes belonging to transmembrane and transporter families, such as ABC proteins and hexose sugar transporters, were functionally characterised and the substrates transported by these proteins rapidly detected using yeast and *Xenopus laevis* oocyte assays [21,24,80]. Genome editing through techniques such as CRISPR is another powerful tool to support gene functional studies in crops, which can also lead to the creation of plants with novel resistance traits (Figure 2). For instance, the *STP13* gene is distributed widely in crops, and the sugar transport function of its protein product remains essential for the successful infection of both biotrophic and necrotrophic pathogens. Thus, a deletion causing loss of function of *STP13* can prevent or slow pathogen infection [26].

Finally, genetic transformation techniques that allow the introduction of genes from wild or unrelated organisms to crop plants constitute ground-breaking technology for crop improvement. For example, using *Agrobacterium tumefaciens*-mediated gene transformation, resistance gene cassettes consisting of both major and durable *APR* genes, sourced from domesticated or wild species, were introduced into wheat for rust resistance [81]. These wheat lines carrying resistance gene combinations were highly resistant and effective against multiple pathogen isolates, as tested in both glasshouse and field conditions [81]. While genetic transformation could assist the development of disease-resistant wheat cultivars, these cultivars would be considered ‘genetically modified organisms’ (GMOs), which are currently not accepted in many countries. To avoid the co-introduction of bacterial DNA molecules into the crop genome, research is underway to exploit a newer peptide-based gene transformation strategy to introduce resistance gene constructs [82].

## Summary

- Adult-plant resistance genes are plant metabolic pathway genes that often provide durable protection against single or multiple pathogens.
- Breeding new cultivars incorporating adult-plant resistance is challenging because only a small number of well-characterised genes are available in elite germplasm.
- Historical genebank accessions provide valuable sources of adult-plant resistance.
- New breeding technologies will allow rapid discovery and introgression of adult-plant resistance genes into modern germplasm to minimise the impact of diseases on yield potential.

## Open Access

Open access for this article was enabled by the participation of University of Queensland in an all-inclusive *Read & Publish* agreement with Portland Press and the Biochemical Society under a transformative agreement with CAUL.

## Abbreviations

ABA: abscisic acid
ABC: adenosine triphosphate-binding cassette
APR: adult-plant resistance
ASR: all-stage resistance
GS: genomic selection
QTL: quantitative trait loci
SNP: single-nucleotide polymorphism
STP: sugar transporter protein
WAK: wall-associated kinase
